# Steroid-induced adrenal insufficiency in children with nephrotic syndrome: a systematic review

**DOI:** 10.1007/s00467-026-07154-4

**Published:** 2026-02-18

**Authors:** Kalliopi Vardaki, Ilianna Maniadaki, Emmanouil Galanakis, Maria Bitsori

**Affiliations:** 1 Departments of Pediatrics and Nephrology, School of Medicine, University of Crete, Heraklion, Greece; 2 Department of Pediatrics, University General Hospital of Heraklion, Heraklion, Greece

**Keywords:** Adrenal insufficiency, Nephrotic syndrome, NS, Steroid-induced complications, ACTH stimulation test

## Abstract

**Background:**

Idiopathic nephrotic syndrome (NS) is the most common glomerular disease in children. While corticosteroids remain the first-line treatment for inducing remission, their prolonged or frequent use can suppress the hypothalamic–pituitary–adrenal (HPA) axis, potentially leading to adrenal insufficiency (AI). However, the true burden of this complication remains poorly understood and inconsistently reported. This study aimed to systematically evaluate the prevalence, diagnostic methods, risk factors, and clinical implications of steroid-induced AI in children with NS.

**Methods:**

A systematic search of Medline was conducted up to October 3, 2025, and the references of relevant publications were also hand-searched for eligible studies. We looked for studies that assessed adrenal function or AI in pediatric patients with NS treated with corticosteroids. Only English-language studies were included; case reports, abstracts, and reviews were excluded. In total, 13 studies involving 516 pediatric patients met the inclusion criteria. The review was conducted in accordance with the Preferred Reporting Items for Systematic Reviews and Meta-Analyses (PRISMA 2020) guidelines and was registered in the PROSPERO database (CRD420251082353, June 27, 2025). Due to methodological heterogeneity, a narrative synthesis was performed.

**Results:**

The reported prevalence of AI ranged from 5.9 to 92.9%, largely influenced by the diagnostic test used and the timing of assessment. Seven different testing methods were identified, with the 2-h ACTH stimulation test demonstrating the highest diagnostic yield. Definitions of AI and cortisol cutoff values varied considerably across studies. AI was more commonly observed in children with frequently relapsing or steroid-dependent NS, as well as in those with prolonged or repeated exposure to corticosteroids. Associations with age were inconsistent. AI was also linked to an increased risk of relapse, particularly during infections. Children with suppressed adrenal function may be at risk of adrenal crisis if not administered stress-dose corticosteroids during periods of physiological stress.

**Conclusion:**

Included studies were mostly small, single-center, and methodologically heterogeneous. There was a lack of consensus on diagnostic criteria and limited long-term follow-up data. Steroid-induced AI is a common and potentially under-recognized complication in children with idiopathic NS, especially in high-risk groups. Due to inconsistent diagnostic practices, the actual prevalence of AI is unclear. There is a critical need for further research, standardized testing protocols, and clinical guidelines. Clinicians may consider screening for AI in children with high cumulative steroid exposure and consider cortisol replacement accordingly.

**Graphical abstract:**

A higher resolution version of the Graphical abstract is available as Supplementary information.

**Figure:**
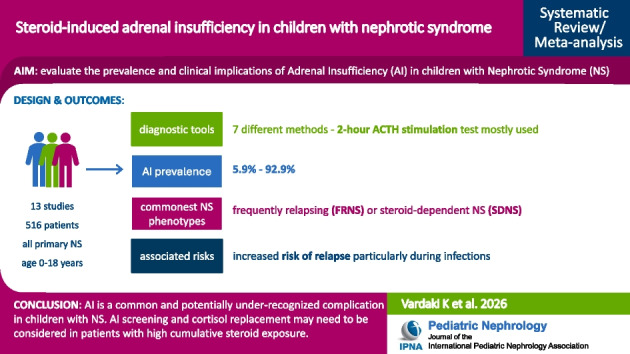

**Supplementary Information:**

The online version contains supplementary material available at 10.1007/s00467-026-07154-4.

## Introduction

Idiopathic nephrotic syndrome (NS) is the most frequent glomerular disease in childhood, characterized by heavy proteinuria, hypoalbuminemia, and edema. The annual incidence of NS in children ranges from 1.15 to 16.9 per 100,000 globally, with minimal change disease (MCD) being the most common histological subtype. Corticosteroids, particularly prednisolone, are the cornerstone of NS treatment, inducing remission in approximately 85–90% of patients within 4–6 weeks of treatment. Among the steroid-sensitive patients, 70–80% will have at least one relapse during follow-up, and up to 50% of these patients will experience frequent relapses or become steroid dependent to maintain remission [1, 2].

Despite its efficacy, prolonged corticosteroid therapy is associated with significant adverse effects, including growth retardation, hypertension, obesity, and suppression of the hypothalamic–pituitary–adrenal (HPA) axis. The glucocorticoid-induced HPA axis suppression can lead to secondary or tertiary adrenal insufficiency (AI), wherein the body’s ability to produce endogenous cortisol is impaired. This suppression can persist for weeks to months after cessation of steroid therapy, with the duration influenced by the cumulative dose and the duration of corticosteroid use.

During the period of prolonged corticosteroid therapy, there is a risk of adrenal crisis, especially during infections or other physiological stress, when cortisol demand increases [3, 4]. The prevalence of corticosteroid-induced AI varies widely, with studies in adult populations reporting rates between 14 and 63%, depending on factors such as steroid dosage, duration of therapy, and individual patient susceptibility [5]. In children with NS, the risk of AI is particularly concerning due to the frequent relapses and the necessity for repeated or prolonged steroid courses. Moreover, AI in this population may be underdiagnosed, as symptoms can be nonspecific and overlap with those of NS or its complications [4, 6].

Currently, there is a gap in evidence regarding several aspects of steroid-induced AI in children with NS, including its incidence across different disease types and demographic backgrounds, the risk factors associated with higher rates, and its relationship with subsequent relapses. Leisti et al. [6] and Abu Bakar et al. [7] suggested that younger children with NS had a higher risk of AI. In contrast, Jaiganesh et al. [8], in their most recent study up to the time of writing of this manuscript, found that older age was a significant factor in developing AI after steroid therapy in NS. Among the available literature, a considerable inconsistency in both the diagnostic methods and the timing of performing the investigations for AI has been recognized. Whereas early morning cortisol levels have been supported by Mantan et al. [8] as a reliable diagnostic tool that may eliminate the need for additional ACTH stimulation testing, Jaiganesh et al. [9] reported them to be inaccurate.

The limited research, along with the heterogeneity in methods and outcomes, has resulted in a lack of consensus on the optimal strategies for screening, diagnosing, and managing AI in patients with NS who are undergoing corticosteroid therapy. The aim of this systematic review was to synthesize the existing evidence, thereby informing clinical practice and guiding future research.

## Materials and methods

### Search strategy and selection criteria

This study was conducted in accordance with the Preferred Reporting Items for Systematic Reviews and Meta-Analyses (PRISMA) guidelines [10], after registration in the PROSPERO database (CRD420251082353, June 27, 2025). A comprehensive search of the MEDLINE (via PubMed) database was conducted, without language or date/year restrictions, to identify articles reporting on the association between idiopathic NS in children and steroid-induced AI. Various search strategies were employed to maximize sensitivity and, finally, the following two algorithms were used: (a) (nephrotic syndrome) AND (adrenal insufficiency) OR (adrenocortical suppression) OR (adrenocortical insufficiency) OR (Synacthen) OR (cortisol) and (b) (adrenal insufficiency) AND (glomerular disease). The final search was run on 03 October 2025. The titles and abstracts of the articles were screened for eligibility. The references of relevant publications were also hand-searched for eligible studies. Studies were included if they enrolled children (≤ 18 years) with NS receiving corticosteroid therapy and reported adrenal function assessments. Studies involving adult populations, secondary or congenital NS, as well as review articles, non-English publications, and duplicate datasets, were excluded. Two authors independently assessed the full-text articles according to the study selection criteria. The included studies were then reviewed in detail for data extraction.

### Data extraction

From each eligible study, data extraction was performed using a standardized electronic form. Information on authors, journal, year of publication, country and region of the study, number of participants, demographics, NS type (steroid sensitive, steroid dependent, frequent relapsing, infrequent relapsing, steroid resistant), NS presentation (first presentation, relapse), steroid agent used, steroid cumulative dose (prednisolone equivalent), steroid course duration, diagnostic method for AI, time since steroid withdrawal to testing for AI, participants who developed AI, participants who did not develop AI, AI participants who relapsed within the first year, non-AI participants who relapsed within the first year, risk factors for AI, and other steroid complications were recorded.

### Statistical analysis

Given the methodological heterogeneity among the included studies, particularly in diagnostic criteria for AI, cortisol assay methods, and timing of HPA axis evaluation, a formal meta-analysis was not performed. Instead, data were synthesized descriptively. Study characteristics and outcomes were summarized in tabular form, and the prevalence of AI was reported as provided in each study. Where possible, results were stratified by NS subtype, steroid exposure, and diagnostic method. Differences and trends across studies were analyzed qualitatively, focusing on patterns of association rather than quantitative pooling. No additional statistical tests or weighting were applied.

## Results

### Search results and included studies

**Fig. 1:**
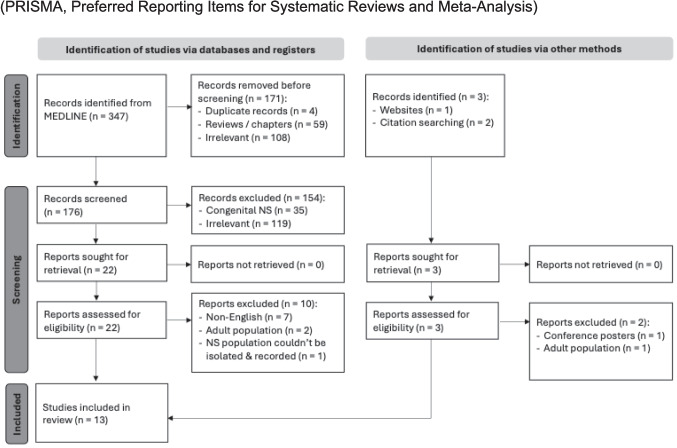
PRISMA flow diagram of the study selection (PRISMA, Preferred Reporting Items for Systematic Reviews and Meta-Analysis)

Study screening and selection are illustrated as a PRISMA flowchart in Fig. 1. A total of 347 records were obtained from database searches, and three records were identified from citation and web searches. Finally, 13 studies were included in this review after the removal of review articles and chapters (*n* = 59), duplicate articles (*n* = 4), irrelevant studies (*n* = 227), articles in non-English languages (*n* = 7), or studies that did not fulfill the selection criteria (*n* = 40). The included studies were published between 1974 and 2025. They were conducted mostly in Europe (6/13, 46.1%), followed by Asia (5/13, 38.5%) and North America (2/13, 15.4%), with none conducted in Africa, Australia, or South America.

### Profile of the participants and treatment characteristics

A total of 516 patients with NS were reviewed with a mean age of 9.1 (0–18) at the time of investigation for AI. All patients were in remission when included, and regarding the time of inclusion, in two studies, the participants were investigated for AI after the first nephrotic presentation [6, 11], in two after any NS relapse [12, 13], and the rest after either the first presentation or a relapse. In the majority of the studies (9/13), patients had completed steroid treatment at the time of testing for AI, and in two studies, testing was performed during low-dose steroids, as part of maintenance therapy [13, 14]. Prednisone was the steroid agent administered in six studies before 1983 [7, 12, 15–18], which was replaced by prednisolone in all the studies conducted later.

The NS subtypes that were investigated in each study are summarized in Table 1. With regard to the definitions used for NS classification, all the researchers followed either the International Study of Kidney Disease in Children (ISKDC) [19] or the KDIGO guidelines [20]. NS was classified as steroid sensitive (SSNS) if remission occurred within 8 weeks of steroids and as steroid resistant (SRNS) if it did not. Steroid dependent (SDNS) was defined as an NS that relapsed during tapering or within 14 days of stopping steroids and a frequently relapsing (FRNS) one that relapsed ≥ 2 times within 6 months or ≥ 4 within a year. Infrequently relapsing NS (IFRNS) was defined as an NS that was sensitive to steroids but did not meet the criteria of FRNS or SDNS. Most of the studies included only SSNS cases that were further classified into IFRNS, FRNS, SDNS, or non-SDNS, with only two studies including children with SRNS [8, 14]. The overall incidence of SSNS and SRNS among the reviewed studies was 87.8% and 12.2%, respectively.

**Table 1:** Nephrotic syndrome subtypes among the studies

Study	Study year	Sample size (*n*)	SSNS (*n*)	SRNS (*n*)	IFRNS/single NS episode (*n*)	FRNS (*n*)	SDNS (*n*)
Zurbrügg R. P. et al. [17]	1974	19	19	0	NA	NA	NA
Leisti S. et al. [18]	1977	27	27	0	17	10	NA
Leisti S. et al. [7]	1977	23	23	0	11	12	0
Moel D. I. et al. [12]	1980	17	17	0	7	10	0
Hansen N. et al. [15]	1982	42	42	0	NA	NA	NA
Leisti S. et al. [16]	1983	47	47	0	NA	NA	NA
Sumboonnanonda A. et al. [21]	1994	14	14	0	NA	NA	NA
Abeyagunawardena A. S. et al. [13]	2007	32	32	0	0	NA	NA
Mantan M. et al. [8]	2018	70	32	38	18	8	6
Abu Bakar K. et al. [6]	2020	37	37	0	9	8	20
Khan T. et al. [11]	2023	56	56	0	NA	NA	NA
Krishna G. M. et al. [14]	2024	52	27	25	6	9	12
Jaiganesh M. et al. [9]	2025	80	80	0	69	NA	NA
**Total *n* (%)**		**516**	**453 (87.8%)**	**63 (12.2%)**	**NA**	**NA**	**NA**

*NS* nephrotic syndrome, *SSNS* steroid sensitive nephrotic syndrome, *SRNS* steroid resistant nephrotic syndrome, *IFRNS* infrequent relapsing nephrotic syndrome, *FRNS* frequent relapsing nephrotic syndrome, *SDNS* steroid-dependent nephrotic syndrome

### Timing and methods for AI diagnosis

According to the inclusion criteria of each study, different time points for AI testing were determined during the treatment with steroids, immediately post-discontinuation, or later. Also, in 5/13 studies (38.5%), the researchers calculated the mean steroid cumulative dose over the last 12 months, as an indicator of steroid exposure. Regarding AI diagnosis, seven different methods were recorded, with discrepancies between the reference cortisol levels used before and after stimulation. One research group used “early morning serum cortisol level” as the exclusive diagnostic tool [8], six used the “2-h ACTH stimulation test” [7, 15–18, 21], one used the “6-h ACTH stimulation test” and the “metyrapone test” [12], one used the “short Synacthen test” [11], one used the “modified low dose Synacthen test” [6, 13], and two used the “long-acting porcine sequence ACTH stimulation test” [9, 14]. Details of the cortisol stimulation technique used in each study are provided in Table 2, and a short description of the stimulation tests used is provided in Table 3. Leisti et al. [18], in addition to using the 2-h ACTH stimulation test, also assessed the plasma cortisol response to insulin administration in a subset of their patients; this component was not included in our analysis to avoid confusion. Cortisol-level units were all converted to µg/dl for consistency purposes.

**Table 2:** Timing and methods for adrenal insufficiency diagnosis

Study	Timing for AI testing	Mean steroid cumulative dose (prednisolone equivalent, last 12 m)	AI diagnostic method	Reference values for AI diagnosis
Zurbrügg R. P. et al. [17]	4–6 days after alternate-day prednisone therapy was discontinued	NA	2-h ACTH stimulation test	2-h plasma cortisol level < 34.3 µg/dl
Leisti S. et al. [18]	4–6 days after alternate-day prednisone therapy was discontinued	NA	2-h ACTH stimulation test	2-h plasma cortisol level < 34.3 µg/dl
Leisti S. et al. [7]	1–12 d after prednisone last dose	NA	2-h ACTH stimulation test	2-h plasma cortisol level < 34 µg/dl
Moel D. I. et al. [12]	1 week after alternate-day prednisone discontinuation	NA	6-h ACTH stimulation test AND metyrapone test	NA
Hansen N. et al. [15]	Immediately after steroid completion	NA	2-h ACTH stimulation test	2-h plasma cortisol level < 30 µg/dl, early morning cortisol level < 8 µg/dl
Leisti S. et al. [16]	1–12 d after prednisone last dose	NA	2-h ACTH stimulation test	2-h plasma cortisol level < 29.4 µg/dl
Sumboonnanonda A. et al. [21]	7 d, 3, 6, and 9 m after discontinuation of prednisolone	NA	2-h ACTH stimulation test	NA
Abeyagunawardena A. S. et al. [13]	On alternate day prednisolone (0.1–1 mg/kg/day, minimum of 12 m and same dose at least for the preceding 3 m)	0.4 mg/kg/day	Modified low-dose Synacthen test	1-h plasma cortisol level < 18 µg/dl
Mantan M. et al. [8]	48 h after the last steroid dose	0.28 mg/kg/day	Early morning serum cortisol level	Early morning cortisol level < 5 µg/dl
Abu Bakar K. et al. [6]	Off steroids for 4–6 w	25.63 mg/m^2^/day	Modified low-dose Synacthen test	1-h plasma cortisol level < 19.94 µg/dl
Khan T. et al. [11]	Immediately after steroid completion	NA	Short Synacthen test	1-h plasma cortisol level < 18 µg/dl, early morning cortisol level < 3 µg/dl
Krishna G. M. et al. [14]	On alternate day prednisolone (< 1 mg/kg/d for at least 8 w or > 2 mg/kg/d for more than 2 w in the past	0.26 mg/kg/day	Long-acting porcine sequence ACTH stimulation test	1-h plasma cortisol level < 18 µg/dl, early morning cortisol level < 5 µg/dl
Jaiganesh M. et al. [9]	Three groups: at 1, 3 or 6-m post-steroid withdrawal	0.23 mg/kg/day	Long-acting porcine sequence ACTH stimulation test	1-h plasma cortisol level < 18 µg/dl, early morning cortisol level < 5 µg/dl

*d* days, *w* weeks, *m* months, *NA* not available, *AI* Adrenal Insufficiency, *ACTH* adrenocorticotropic hormone

**Table 3:** Cortisol stimulation techniques that were used for adrenal insufficiency diagnosis

Type of technique	Characteristics
2-h ACTH stimulation test	iv bolus of ACTH analogue (0.25 mg/1.73 m^2^) given and blood samples for fluorometric determination of plasma cortisol taken at 1, 2, and 3 h after the injection [25]
6-h ACTH stimulation test	iv bolus of ACTH analogue given and blood samples for fluorometric determination of plasma cortisol taken at 1, 4, and 6 h after the injection [25]
Metyrapone test	Metyrapone 30 mg/kg administered at 12 midnight and cortisol and 11-deoxycortisol concentrations measured at 8:00 the following morning. Postmetyrapone 11-deoxycortisol concentrations below 7.25 µg/dl at 8:00 are consistent with a diagnosis of adrenal insufficiency [26, 27]
Short Synacthen test	iv bolus of ACTH analogue (250 µg) administered and blood sample for serum cortisol taken at 60 min. Peak cortisol level < 18 µg/dl in post-Tetracosactrin sample are defined as suppressed HPA axis [28]
Modified low-dose Synacthen test	iv bolus of ACTH analogue (0.5 µg) administered and blood samples drawn at 0, 10, 20, 30, 40, 50 and 60 min. A peak plasma cortisol concentration of < 18 µg/dl indicative of abnormal adrenal function [29]
Long-acting porcine sequence ACTH stimulation test	im 25 IU of long-acting porcine sequence ACTH injected, and serum cortisol sample taken after 1 h. All patients with 1 h post-ACTH cortisol < 18.0 µg/dl are considered as having AI [30]

*iv* intravenous, *im* intramuscular, *ACTH* adrenocorticotropic hormone

### Prevalence of AI

As previously mentioned, the AI assessment techniques and the reference cortisol thresholds varied across the 13 studies included. Hence, the prevalence of AI could not be calculated cohesively, with some of the studies showing different results even between the two or three methods used in specific populations at the same time. Table 4 summarizes the AI prevalence reported in each study, considering the various assessment techniques. The highest AI prevalence (92.9%) was found by Hansen et al. [15] using the 2-h ACTH stimulation test, and the lowest (5.9%) by Moel et al. [12] using the 6-h ACTH stimulation test, with both having examined patients with SSNS after the steroid treatment completion. In four studies, other steroid complications were recorded along with the AI quantification: obesity, hypertension, cushingoid appearance, hirsutism, myalgia/weakness, and cataract. The number of children who developed signs of steroid toxicity (ST) compared to those diagnosed with AI (ST/AI) was 4/5 [21], 23/28 [8], 23/13 [6], and 28/36 [19], respectively.

**Table 4:** Prevalence of adrenal insufficiency among the studies and diagnostic techniques used

Study	Sample size (*n*)	Prevalence (%)	Method for AI diagnosis
Zurbrügg RP et al. [17]	19	31.6	2-h ACTH stimulation test
Leisti S et al. [18]	27	14.8	Morning cortisol level
		37.0	2-h ACTH stimulation test
Leisti S et al. [7]	23	47.8	2-h ACTH stimulation test
Moel DI et al. [12]	17	58.8	Morning cortisol level
		5.9	6-h ACTH stimulation test
		6.3	Metyrapone test
Hansen N et al. [15]	42	52.4	Morning cortisol level
		92.9	2-h ACTH stimulation test
Leisti S et al. [16]	47	50.8	2-h ACTH stimulation test
Sumboonnanonda A. et al. [21]	14	36	2-h ACTH stimulation test (at 7 d and 3 m)
Abeyagunawardena A. S. et al. [13]	32	62.5	Modified low-dose Synacthen test
Mantan M. et al. [8]	70	40	Morning cortisol level
Abu Bakar K. et al. [6]	37	35.1	Modified low-dose Synacthten test
Khan T. et al. [11]	56	69.6	Morning cortisol level
		91.1	Short Synacthen test
Krishna G. M. et al. [14]	52	50	Morning cortisol level
		69.2	Long-acting porcine sequence ACTH stimulation test
Jaiganesh M. et al. [9]	80	10	Morning cortisol level
		32.5	Long-acting porcine sequence ACTH stimulation test

*AI* Adrenal Insufficiency, *ACTH* adrenocorticotropic hormone

### AI variations among the NS subtypes and associated risk factors

The prevalence of AI among the different NS subtypes was assessed in six studies. Three research groups identified FRNS as the one most commonly associated with AI, with frequencies of 63.6% [7], 100% [12], and 77.8% [14], respectively. Moel et al. [12] and Mantan et al. [8] showed that plasma cortisol was significantly lower in frequently relapsing individuals compared to the infrequent relapsers (*p* < 0.05). Jaiganesh et al. [9] reported IFRNS as the most frequent subtype (84.6%), whereas Mantan et al. [8] found the highest AI prevalence in SRNS (57%) with FRNS to follow (28%), and Abu Bakar et al. [6] in SDNS (76.9%). Three of the studies compared relapses within the first year between the patients who developed AI and those who did not, all showing a higher incidence of relapse in the AI group. Leisti et al. [16] noted that all the patients with AI belonged to the group whose first relapse occurred within a year (*p* < 0.10) [7]. Similarly, Leisti et al. [16] and Abeyagunawardena et al. [13] showed a significant difference in the prevalence of relapses during the first year between the AI and non-AI groups, with 88% vs. 79% (*p* < 0.05) and 100% vs. 58% (*p* = 0.01), respectively.

In seven of the thirteen studies, risk factors associated with AI were recorded. Two studies found a significant correlation between AI and young age [6, 7], while another found a correlation with older age [9]. In two studies, higher/longer exposure to steroids was marked as a statistically significant risk factor for AI [8, 14]. Table 5 provides a detailed summary of the risk factors, along with the most frequent NS subtypes associated with AI and the 12-month relapse incidence for both AI and non-AI patients across the reviewed studies.

**Table 5:** Nephrotic syndrome subtypes, relapse incidence within the first year and risk factors for adrenal insufficiency

Study	NS subtype with highest AI frequency	AI/non-AI relapse frequency within first year (%)	RISK factors for AI
Zurbrügg R. P. et al. [17]	NA	NA	NA
Leisti S. et al. [18]	NA	NA	No significant correlation with several factors (age, duration of illness, frequency of relapses, hypoalbuminemia, proteinuria)
Leisti S. et al. [7]	FRNS (63.6%)	100/46	• Higher premedication proteinuria (*p* < 0.10) • Young age (*p* < 0.05) • URTI (*p* < 0.06)
Moel D. I. et al. [12]	FRNS (100%)	NA	NA
Hansen N. et al. [15]	NA	NA	NA
Leisti S. et al. [16]	NA	88/79	NA
Sumboonnanonda A. et al. [21]	NA	NA	NA
Abeyagunawardena A. S. et al. [13]	NA	100/58	NA
Mantan M. et al. [8]	SRNS (57%)	NA	• Significantly higher mean prednisolone doses (0.28 mg/kg/d vs. 0.089 mg/kg/d) (p 0.00) • Longer steroid therapy duration (p 0.001)
Abu Bakar K. et al. [6]	SDNS (76.9%)	NA	• Younger age at diagnosis (mean, 2.54 years) (95% CI, 0.567–0.990) • SDNS (6 times more likely to develop AI) (95% CI, 1.06–29.34)
Khan T. et al. [11]	NA	NA	• Divided vs. single prednisolone dose (*p* = 0.02)
Krishna G. M. et al. [14]	FRNS (77.8%)	NA	• Cumulative steroid intake of > 0.22 mg/kg/day (*p* < 0.002)
Jaiganesh M. et al. [9]	IFRSN (84.6%)	NA	• Older children and children with older age at diagnosis (*p* < 0.05) • Lower basal cortisol level (*p* < 0.05)

*NA* not available, *NS* nephrotic syndrome, *AI* adrenal insufficiency, *FRNS* frequent relapsing nephrotic syndrome, *SRNS* steroid resistance nephrotic syndrome, *SDNS* steroid-dependent nephrotic syndrome, *IFRNS* infrequent relapsing nephrotic syndrome, *URTI* upper respiratory tract infection

## Discussion

Over the past 50 years, only a limited number of studies have focused on various aspects of steroid-induced AI in children with NS. In this review, we systematically appraised the available evidence, revealing considerable heterogeneity in diagnostic and management approaches. Despite five decades of sporadic investigation, most data have emerged only recently and remain inconclusive, underlining the need for further standardized and methodologically consistent research to better define the diagnostic criteria, the true prevalence, and risk factors of AI.

Although a high prevalence of AI in pediatric NS, particularly in cases requiring high cumulative steroid doses, is anticipated, our analysis indicates that the strength of this association remains inconsistently demonstrated across the literature. The reported prevalence varies widely (5.9–92.9%), largely reflecting methodological heterogeneity among studies, rather than true biological divergence. At least seven different diagnostic protocols were employed, resulting in substantially discrepant findings. For example, Hansen et al. [15] reported the highest AI rate using a 2-h ACTH stimulation test, while Moel et al. [12] found the lowest prevalence using a 6-h ACTH test. The choice of diagnostic test appears to substantially influence AI detection, a point emphasized by Jaiganesh et al. [9], who noted that normal basal cortisol levels were not predictive of normal ACTH responses in nearly 77% of affected children. Although the short Synacthen test has been considered the clinical gold standard for evaluating adrenal function since the 1970 s, even research conducted in the past decade has shown inconsistency in diagnostic approaches. Notably, the two most recent studies [9, 14] used the long-acting porcine sequence ACTH stimulation test, which is no longer considered valid or standardized, due to the limited availability of Synacthen in certain regions.

This variability extends to the timing of testing, with studies assessing AI during ongoing steroid therapy, immediately after steroid discontinuation, or weeks thereafter. The influence of steroid pharmacokinetics and recovery dynamics of the HPA axis likely contributes to inconsistent detection rates. Furthermore, no universally accepted threshold for cortisol insufficiency was used across studies, complicating comparative analysis and pooled prevalence estimates.

The heterogeneity in AI prevalence is also mirrored in the differential risk across NS subtypes. While most studies focused exclusively on steroid-sensitive NS, some included children with steroid-resistant, frequently relapsing, or steroid-dependent courses. Several studies identified FRNS as the subtype most associated with AI, with reported frequencies as high as 100% [12]. Others found SDNS [6] or SRNS [8] to be the most affected, while IFRNS showed the lowest association [9]. This inconsistency may be partially attributed to differences in patient selection, steroid dosing histories, and duration of therapy at the time of testing.

Importantly, frequent relapsers appear to be particularly vulnerable to HPA axis suppression. Moel et al. [12] and Mantan et al. [8] both reported significantly lower plasma cortisol levels in FRNS patients compared to IFRNS patients (*p* < 0.05). Moreover, Leisti et al. [7] suggested that younger children, who tend to relapse more frequently, may be particularly sensitive to steroid-induced suppression due to surface area-based dosing. Abeyagunawardena et al. [13] and Leisti et al. [16] demonstrated significantly higher 12-month relapse rates in AI patients compared to non-AI peers. This raises an important clinical question as to whether AI independently increases relapse risk or is merely a marker of prior disease severity and steroid burden.

Abeyagunawardena et al. [13] argue that steroid-sparing agents (e.g., levamisole, ciclosporin) were preferentially used in severe cases, thus mitigating confounding by disease severity. They also propose a plausible immunologic mechanism: HPA suppression may blunt the body’s stress response, leading to unchecked cytokine surges during infections, a known relapse trigger in NS. Khan et al. [11] further support this hypothesis, citing randomized controlled trials in South Asia where steroid “stress dosing” during upper respiratory infections reduced relapse frequency [22, 23]. In contrast, the UK-based PREDNOS 2 trial [24] found no significant benefit of a 6-day prednisolone course during upper respiratory tract infections, possibly reflecting lower cumulative steroid exposure and reduced adrenal suppression due to greater use of steroid-sparing therapies than in the Asian subcontinent.

The potential link between HPA suppression and disease relapse was first reported as early as the late 1970s to early 80s. Leisti et al. [7, 16, 18], in their consecutive studies, suggest routine ACTH testing in high-risk patients and even temporary cortisol substitution in those with severe suppression to reduce relapse risk and prevent adrenal crises. These reports did not receive due consideration at the time, perhaps due to complicated methodology and indirect results, which could not safely prove causality. Krishna et al. [14] echo this recommendation, proposing stress-dose steroids during acute illness or surgery for patients with cumulative steroid exposure > 0.22 mg/kg/day.

Despite the growing interest in this topic, several key questions remain unanswered. First, there is no consensus on the optimal timing or preferred method for AI assessment in NS patients. Although morning cortisol has been proposed as the initial step for evaluating HPA axis integrity [3, 31], it appears to be inadequate as a standalone tool. Moreover, the ACTH stimulation tests used in clinical practice differ considerably in design, availability, and interpretive thresholds, and debate regarding which test most accurately detects AI is ongoing [3]. Second, the prevalence of AI across NS subtypes remains unclear due to inconsistent subgroup analysis and limited inclusion of patients with SRNS, SDNS, and IFRNS. Third, the clinical significance of subclinical AI, particularly its impact on relapse risk and morbidity, needs to be better defined through prospective longitudinal studies.

Also, strategies to minimize HPA axis suppression deserve further exploration. These include evaluating the safety and efficacy of alternate-day steroid regimens, early transitioning to steroid-sparing agents, and personalizing tapering protocols. As emphasized in the NICE guideline 2024 [32], during the steroid tapering phase, when AI-related adverse events are most likely to occur, there is insufficient evidence on how best to taper in order to ensure timely recovery of adrenal function. Future studies should also examine the role of genetic and environmental factors in modulating steroid responsiveness and HPA recovery, as suggested by the differing outcomes of the PREDNOS 2 trial [24] compared with South Asian studies [11, 22, 23].

Finally, joint Nephrology–Endocrinology guidelines are needed to provide standardized recommendations for AI evaluation and management in patients with NS. It is well known that during periods of adrenal insufficiency, patients are at increased risk of adrenal crisis, particularly during infections, major surgeries, or other physiological stressors. The current treatment approach for any first episode of childhood NS includes at least 4 weeks of prednisone/prednisolone 60 mg/m^2^/day, followed by a quick tapering. This cumulative steroid exposure invariably meets the criteria for long-term glucocorticoid therapy (treatment duration > 3–4 weeks with doses exceeding the physiologic cortisol secretion (hydrocortisone 10 mg/m^2^/day or prednisolone 2.5 mg/m^2^/day). According to the recent joint clinical guideline from the European Society of Endocrinology and the Endocrine Society for glucocorticoid-induced adrenal insufficiency [33], all patients with NS receiving treatment can be expected to have suppressed adrenal function. The guideline further recommends that all patients with current or recent glucocorticoid use who have not undergone biochemical testing to rule out glucocorticoid-induced AI should receive stress-dose coverage during periods of stress, along with appropriate patient education to raise awareness of when stress dosing is required. Further research is required to clarify whether cortisol replacement therapy should be initiated in confirmed AI, and to define the optimal dosing regimen and duration.

In light of the current evidence and taking into account the endocrinology practice guidelines [3, 33], we can only summarize pragmatic recommendations based on early identification of children with NS at high risk for AI, which is the only point of convergence of the reviewed studies (Table 6). The recommendations should be adjusted to the local protocols for steroid toxicity prevention and AI management. 

**Table 6:** Recommendations for adrenal insufficiency management

Who to screen	• FRNS
	• SDNS
	• Prolonged steroid exposure (> 4 weeks) beyond the initial episode
	• Cumulative steroid doses exceeding hydrocortisone 10 mg/m^2^/day or prednisolone 2.5 mg/m^2^/day
	• Frequent relapses within the first year
How to screen	• A morning serum cortisol level (between 8:00 and 9:00 AM) after holding glucocorticoid dose for at least 24 h
	• Interpretation of morning serum cortisol level: – > 10 µg/dL possible restored HPA axis – 5–10 µg/dL indeterminate levels – < 5 µg/dL HPA axis not yet restored
	• Additional Synacthen test when levels indeterminate or falsely low
When to consider adrenal crisis	• Children with confirmed AI during stress (acute illness, surgery, or trauma)
	• Children with current or recent steroids use not yet tested for AI during stress
	• Children on steroids presenting with hemodynamic instability, hypoglycemia, acute abdominal symptoms
	• Stress-dose: hydrocortisone 100 mg/m^2^
When to consider cortisol replacement	• In all patients with suspected AI
	• Physiologic-dose: 8–10 mg/m^2^/day in 2–3 divided doses)
	• Until normal HPA function is demonstrated with reassessment every 1–3 months
Long-term management	• Gradual glucocorticoid tapering with careful monitoring for AI manifestations
	• If AI symptoms occur, continue steroids until HPA recovery
	• Prefer hydrocortisone over prednisolone during the final tapering phase
	• Early initiation of glucocorticoid-sparing agents in recurrent or steroid-dependent cases

*FRNS* frequent relapsing nephrotic syndrome, *SDNS* steroid-dependent nephrotic syndrome, *HPA axis* hypothalamic–pituitary–adrenal axis, *ACTH* adrenocorticotropic hormone, *AI* adrenal insufficiency

Children with FRNS, SDNS, prolonged steroid exposure (> 4 weeks) beyond the initial episode, cumulative steroid doses exceeding the normal cortisol secretion (hydrocortisone 10 mg/m^2^/day) or frequent relapses within the first year, are at high risk for AI and may undergo HPA axis evaluation. A morning serum cortisol level, measured between 8:00 and 9:00 AM, after holding the glucocorticoid dose for at least 24 h and only after reaching the range of a physiologic equivalent daily dose, seems to be a reasonable screening approach. A Synacthen test may be further required when the cortisol level is low or indeterminate or in conditions where the total serum cortisol is falsely low. Immediate initiation of stress-dose parenteral glucocorticoids and fluid resuscitation is recommended for children with confirmed AI or for those with current or recent glucocorticoid use who have not yet been tested for glucocorticoid-induced AI during periods of acute illness, surgery, or trauma. Adrenal crisis might also occur in patients taking oral supraphysiologic doses of glucocorticoids if drug availability suddenly decreases due to missed doses or gastroenteritis. Stress-dose steroids warrant consideration in patients presenting with hemodynamic instability, hypoglycemia, or acute abdominal symptoms. It is advised that clinicians and families remain aware of the potential AI features and that rescue hydrocortisone for stress dosing (hydrocortisone 100 mg/m^2^) be prescribed when appropriate. Physiologic daily dose replacement (hydrocortisone 8–10 mg/m^2^/day in 2–3 divided doses) is advocated in all patients with suspected AI until normal HPA function is demonstrated. In practice, this may require 3–6 months of follow-up, though individual recovery times vary. Reassessment could be scheduled every 1–3 months until recovery is documented. A final tapering phase with hydrocortisone, which is preferred over prednisolone for its shorter half-life to facilitate faster recovery of adrenal function, might be considered for frequent relapsers or steroid-dependent nephrotic patients. If symptoms of AI occur, the glucocorticoid regimen could be reinstated and not discontinued until recovery of the HPA axis is documented. Early initiation of glucocorticoid-sparing agents, such as levamisole or cyclosporine, may help minimize cumulative steroid exposure and thereby mitigate the risk of adrenal suppression in FRNS or SDNS. Our recommendation should be interpreted as a pragmatic preventive strategy, consistent with contemporary clinical practice aimed at reducing glucocorticoid toxicity, rather than a definitive evidence-based intervention. Future studies are required to assess the impact of such therapeutic adjustments on both relapse control and adrenal function recovery in this population.

This systematic review has several limitations. The literature search was limited to a single database (MEDLINE), and non-English language articles were excluded due to resource constraints. Also, considerable heterogeneity among the included studies in design, diagnostic criteria for AI, and outcome reporting precluded the quantitative meta-analysis. Some of the studies were conducted more than 2 decades ago, and the evolution in corticosteroid dosing protocols, diagnostic assays, and clinical definitions since then limits direct comparability with more recent data. These factors should be considered when interpreting the results and in planning future research.

## Conclusion

Given the high clinical stakes associated with undiagnosed AI and the observed heterogeneity in both its detection and prevalence, a standardized approach to screening, prevention, and treatment is warranted. Future prospective studies should define optimal diagnostic approaches, clarify recovery timelines of adrenal function, and establish evidence-based guidelines for corticosteroid tapering and replacement. In the interim, individualized management based on risk stratification and clinical judgment remains essential to minimize the risk of adrenal crisis and optimize outcomes for children with NS.

## Supplementary Information

Below is the link to the electronic supplementary material.

ESM1 (PDF 270 KB)

ESM2 (PPTX 74.4 KB)
