# Supplementary material for: Steroid-induced adrenal insufficiency in children with nephrotic syndrome: a systematic review
Source: Pediatr Nephrol. 2026 Feb 18;41(10):3303–13. doi: 10.1007/s00467-026-07154-4 (PMC13481571; doi:10.1007/s00467-026-07154-4)
Supplement: Supplementary file 1 [file 467_2026_7154_MOESM1_ESM.pdf]

PRISMA 2020 Checklist

Section and Topic	Item #	Checklist item	Location where item is reported
**TITLE**			
Title	1	Identify the report as a systematic review.<br>"Steroid Induced Adrenal Insufficiency in Children with Nephrotic Syndrome: A Systematic Review"	Page 1
**ABSTRACT**			
Abstract	2	See the PRISMA 2020 for Abstracts checklist.<br><br>**Background**<br>Idiopathic nephrotic syndrome (NS) is the most common glomerular disease in children. While corticosteroids remain the first-line treatment for inducing remission, their prolonged or frequent use can suppress the hypothalamic-pituitary-adrenal (HPA) axis, potentially leading to adrenal insufficiency (AI). However, the true burden of this complication remains poorly understood and inconsistently reported. This study aimed to systematically evaluate the prevalence, diagnostic methods, risk factors, and clinical implications of steroid-induced AI in children with NS.<br><br>**Methods**<br>A systematic search of Medline was conducted up to the 1st of September 2025 and references of relevant reviews were also hand-searched for eligible studies. We looked for studies that assessed adrenal function or AI in pediatric patients with NS treated with corticosteroids. Only English-language studies were included; case reports, abstracts, and reviews were excluded. In total, 11 studies involving 470 pediatric patients met the inclusion criteria. The review was conducted in accordance with the Preferred Reporting Items for Systematic Reviews and Meta-Analyses (PRISMA 2020) guidelines. Due to methodological heterogeneity, a narrative synthesis was performed.<br><br>**Results**<br>The reported prevalence of AI ranged from 5.9% to 92.9%, largely influenced by the diagnostic test used and the timing of assessment. Seven different testing methods were identified, with the 2-hour ACTH stimulation test demonstrating the highest diagnostic yield. Definitions of AI and cortisol cutoff values varied considerably across studies. AI was more commonly observed in children with frequently relapsing or steroid-dependent NS, as well as in those with prolonged or repeated exposure to corticosteroids. Associations with age were inconsistent. AI was also linked to an increased risk of relapse, particularly during infections. Children with suppressed adrenal function may be at risk of adrenal crisis if not administered stress-dose corticosteroids during periods of physiological stress.<br><br>**Conclusion**<br>Included studies were mostly small, single-center and methodologically heterogeneous. There was a lack of consensus on diagnostic criteria and limited long-term follow-up data. Steroid-induced AI is a common and potentially under-recognised complication in children with idiopathic NS, especially in high-risk groups. Due to inconsistent diagnostic practices, the actual prevalence of AI is unclear. There is a critical need for further research, standardised testing protocols and clinical guidelines. Clinicians may consider screening for AI in children with high cumulative steroid exposure and consider cortisol replacement accordingly.	Page 2
**INTRODUCTION**			
Rationale	3	Describe the rationale for the review in the context of existing knowledge.	Page 3

PRISMA

PRISMA 2020 Checklist

Section and Topic	Item #	Checklist item	Location where item is reported
		Currently, there is a gap in evidence regarding several aspects of steroid induced AI in children with NS, including its incidence across different disease types and demographic backgrounds, the risk factors associated with higher rates, and its relationship with subsequent relapses. Leisti S et al. and Abu Bakar K et al. suggested that younger children with NS had a higher risk of AI. In contrast, Jaiganesh M et al., in the most recent study up to the time of writing this manuscript, found that older age was a significant factor for developing AI after steroid therapy in NS. Among the available literature, a considerable inconsistency on both the diagnostic methods and the timing of performing the investigation for AI has been recognized. Whereas early morning cortisol levels have been supported from Mantan M et al. as a reliable diagnostic tool that may eliminate the need for additional ACTH stimulation testing, Jaiganesh M et al. reported them to be inaccurate [8,9]. The limited research, along with the heterogeneity in methods and outcomes, has resulted in a lack of consensus on the optimal strategies for screening, diagnosing, and managing adrenal insufficiency in patients with nephrotic syndrome undergoing corticosteroid therapy.	
Objectives	4	Provide an explicit statement of the objective(s) or question(s) the review addresses.<br><br>The aim of this systematic review was to synthesize the existing evidence and thereby informing clinical practice and guiding future research on the optimal strategies for screening, diagnosing, and managing adrenal insufficiency in patients with nephrotic syndrome undergoing corticosteroid therapy.	Page 4
**METHODS**			
Eligibility criteria	5	Specify the inclusion and exclusion criteria for the review and how studies were grouped for the syntheses.<br><br>A comprehensive search of Medline database was conducted, without language or date/year restriction, to identify articles reporting on the association between idiopathic nephrotic syndrome in children and steroid induced adrenal insufficiency. The titles and abstracts of the articles were screened for eligibility. Review articles, duplicate studies and studies in non-English language were excluded. Studies in adult populations and in children with non-idiopathic NS (congenital or secondary) were also excluded.	Page 4
Information sources	6	Specify all databases, registers, websites, organisations, reference lists and other sources searched or consulted to identify studies. Specify the date when each source was last searched or consulted.<br><br>A comprehensive search of Medline database was conducted, along with web and citation searching. Last search was updated on the 3rd of October 2025.	Page 4
Search strategy	7	Present the full search strategies for all databases, registers and websites, including any filters and limits used.<br><br>Various search strategies were employed to maximize sensitivity and, finally, the following two algorithms were used in Medline: a) (nephrotic syndrome) AND (adrenal insufficiency) OR (adrenocortical suppression) OR (adrenocortical insufficiency) OR (synacthen) OR (cortisol)), b) (adrenal insufficiency) AND (glomerular disease). Additionally, citations of the relevant articles & reviews were checked for eligible studies and the topic 'steroid-induced adrenal insufficiency in children with nephrotic syndrome' was searched on the web.	Page 4
Selection process	8	Specify the methods used to decide whether a study met the inclusion criteria of the review, including how many reviewers screened each record and each report retrieved, whether they worked independently, and if applicable, details of automation tools used in the process.<br><br>The studies arose from the search strategies were checked one by one. Two authors (KV, MM) independently reviewed titles and abstracts of the records and discussed inconsistencies until consensus was obtained.	Page 4
Data collection process	9	Specify the methods used to collect data from reports, including how many reviewers collected data from each report, whether they worked independently, any processes for obtaining or confirming data from study investigators, and if applicable, details of automation tools used in the process.<br><br>From each eligible study, data extraction was performed in a standardized electronic form by the first author (KV).	Page 4

PRISMA 2020 Checklist

Section and Topic	Item #	Checklist item	Location where item is reported
Data items	10a	List and define all outcomes for which data were sought. Specify whether all results that were compatible with each outcome domain in each study were sought (e.g. for all measures, time points, analyses), and if not, the methods used to decide which results to collect.<br><br>List of ooutcomes sought: NS type (steroid sensitive, steroid dependent, frequent relapsing, infrequent relapsing, steroid resistant), NS presentation (first presentation, relapse), steroid agent used, steroid cumulative dose (prednisolone equivalent), steroids course duration, diagnostic method for AI, time since steroid withdrawal testing for AI, participants who developed AI, participants who did not develop AI, AI participants who relapsed within 1st year, non-AI participants who relapsed within 1st year, risk factors for AI, other steroids complications.<br>For outcomes/values that were not available in certain studies, NA (=not available) was noted under the respective category. For values that were given in inconsistent units, adjustment was performed aiming for higher homogeneity.	Page 4
	10b	List and define all other variables for which data were sought (e.g. participant and intervention characteristics, funding sources). Describe any assumptions made about any missing or unclear information.<br><br>Other variables for which data were sought: Information on authors, journal, year of publication, country and region of the study, number of participants, demographics. No assumptions made	Page 4
Study risk of bias assessment	11	Specify the methods used to assess risk of bias in the included studies, including details of the tool(s) used, how many reviewers assessed each study and whether they worked independently, and if applicable, details of automation tools used in the process.<br><br>Risk of bias not assessed due to missing outcomes/results	Page 4
Effect measures	12	Specify for each outcome the effect measure(s) (e.g. risk ratio, mean difference) used in the synthesis or presentation of results.<br><br>We aimed for a Quantitative Synthesis (Meta-analysis), but studies were not sufficiently similar in design, participants, interventions, and outcomes. So finally, Qualitative Synthesis was performed for all the outcomes	Page 4
Synthesis methods	13a	Describe the processes used to decide which studies were eligible for each synthesis (e.g. tabulating the study intervention characteristics and comparing against the planned groups for each synthesis (item #5)).<br><br>Qualitative Synthesis was performed for all the outcomes due to inadequate compatibility of the studies parameters.	Page 4
	13b	Describe any methods required to prepare the data for presentation or synthesis, such as handling of missing summary statistics, or data conversions.<br>Qualitative Synthesis was performed for all the outcomes due to inadequate compatibility of the studies parameters.	Page 4
	13c	Describe any methods used to tabulate or visually display results of individual studies and syntheses.<br>Qualitative Synthesis was performed for all the outcomes due to inadequate compatibility of the studies parameters.	Page 4
	13d	Describe any methods used to synthesize results and provide a rationale for the choice(s). If meta-analysis was performed, describe the model(s), method(s) to identify the presence and extent of statistical heterogeneity, and software package(s) used.<br>Qualitative Synthesis was performed for all the outcomes due to inadequate compatibility of the studies parameters.	Page 4
	13e	Describe any methods used to explore possible causes of heterogeneity among study results (e.g. subgroup analysis, meta-regression).<br>Qualitative Synthesis was performed for all the outcomes due to inadequate compatibility of the studies parameters.	Page 4
	13f	Describe any sensitivity analyses conducted to assess robustness of the synthesized results.<br>Qualitative Synthesis was performed for all the outcomes due to inadequate compatibility of the studies parameters.	Page 4
Reporting bias assessment	14	Describe any methods used to assess risk of bias due to missing results in a synthesis (arising from reporting biases).<br>Risk of bias not assessed due to missing outcomes/results	Page 4
Certainty assessment	15	Describe any methods used to assess certainty (or confidence) in the body of evidence for an outcome.<br>Certainty not assessed due to missing outcomes/results	Page 4

PRISMA 2020 Checklist

Section and Topic	Item #	Checklist item	Location where item is reported
**RESULTS**			
Study selection	16a	Describe the results of the search and selection process, from the number of records identified in the search to the number of studies included in the review, ideally using a flow diagram.<br><br>A total of 347 records were obtained from database searches, and 3 records were identified from citation and web searches. Finally, 11 studies were included in this review after the removal of review articles and chapters (n = 59), duplicate articles (n = 4), irrelevant studies (n = 227), articles in non-English language (n = 7) or studies that did not fulfil the selection criteria (n = 40).	Page 5
	16b	Cite studies that might appear to meet the inclusion criteria, but which were excluded, and explain why they were excluded.<br><br>PMID 13824833 - language<br>PMID 5520758 - language<br>PMID 4368380 - article not available<br>PMID 197476 - article not available<br>PMID 6146741 - language<br>PMID 2168798 - language<br>PMID 7624163 - language<br>PMID 10361421 - language<br>PMID 14736978 - adult population<br>PMID 28888057 - NS population couldn't be isolated and recorded<br>PMID 28098040 - language<br>PMID 30557879 - adult population ≥20 years<br>PMID 31060510 - adult population	NA
Study characteristics	17	Cite each included study and present its characteristics.<br><br>PMID 71603 - prospective study<br>PMID 6250751- prospective study<br>PMID 6286520 - prospective study<br>PMID 6312005 - retrospective study<br>PMID 7798846 - retrospective study<br>PMID 17284479 - prospective clinical trial<br>PMID 29962670 - prospective study<br>PMID 32351921 - prospective study<br>PMID 37335578 - prospective Randomised Clinical Trial<br>PMID 38253887 - prospective study<br>PMID 39792254 - prospective study	Page 16
Risk of bias in studies	18	Present assessments of risk of bias for each included study.<br>Risk of bias not assessed due to missing outcomes/results	Page 5
Results of individual studies	19	For all outcomes, present, for each study: (a) summary statistics for each group (where appropriate) and (b) an effect estimate and its precision (e.g. confidence/credible interval), ideally using structured tables or plots.<br><br>A total of 470 patients with NS were reviewed with a mean age of 8.2 (0-18) at the time of investigation for AI. All the patients were in remission when included and, regarding the time of inclusion, in 2 studies the participants were investigated for AI after the first nephrotic presentation [6,11], in 2 after any NS relapse [12, 13] and in the rest after either the first presentation or a relapse. In the majority of the studies (9/11), patients had completed	Page 5

PRISMA 2020 Checklist

Section and Topic	Item #	Checklist item	Location where item is reported
		steroid treatment at the time of testing for AI and in two studies, testing was performed during low-dose steroids, as part of maintenance therapy [13,14]. Prednisone was the steroid agent administrated in 4 studies before 1983 [7, 12, 15, 16], whereas that was replaced by prednisolone in all the studies conducted later.<br><br>The NS subtypes that were investigated in each study are summarized in Table 1. With regards to the definitions used for NS classification, all the researchers followed either the International Study of Kidney Disease in Children (I.S.K.D.C.) [17] or the KDIGO guidelines [18]. NS was classified as steroid sensitive (SSNS) if remission occurred within 8 weeks of steroids and as steroid resistant (SRNS) if it did not. Steroid dependent (SDNS) was defined as the NS which relapsed during tapering or within 14 days of stopping steroids and frequently relapsing (FRNS) the one that relapsed ≥2 times within 6 months or ≥4 within a year. Infrequently relapsing NS (IFRNS) was defined as the NS that is sensitive to steroids but did not meet the criteria of FRNS or SDNS. Most of the studies included only SSNS cases that were further classified into IFRNS, FRNS, SDNS or not, with only 2 studies having also included children with SRNS [8,14]. The overall incidence of SSNS and SRNS among the reviewed studies was 86.6% and 13.4% respectively.<br><br>Meta-analysis not performed. Qualitative Synthesis was performed for all the outcomes due to inadequate compatibility of the studies parameters.	
Results of syntheses	20a	For each synthesis, briefly summarise the characteristics and risk of bias among contributing studies.<br><br>The included studies were published between 1977 and 2025. They conducted mostly in Asia (5/11, 45.5%), followed by Europe (4/11, 36.4%) and North America (2/11, 18.2%), with none conducted in Africa, Australia, or South America.<br><br>Risk of bias not assessed due to missing outcomes/results	Page 5
	20b	Present results of all statistical syntheses conducted. If meta-analysis was done, present for each the summary estimate and its precision (e.g. confidence/credible interval) and measures of statistical heterogeneity. If comparing groups, describe the direction of the effect.<br><br>Timing and Methods for AI Diagnosis<br>According to the inclusion criteria of each study, different time points for AI testing were determined; during the treatment with steroids, immediately post discontinuation or later. Also, in 5/11 studies (45.5%) the researchers calculated the mean steroid cumulative dose over the last 12 months, as an indicator of steroid exposure. Regarding AI diagnosis, seven different methods were recorded with discrepancy between the reference cortisol level used both before and after stimulation. One research group used 'early morning serum cortisol level' as the exclusive diagnostic tool [8], four used '2-hour ACTH stimulation test' [7,15,16,19], one used '6-hour ACTH stimulation test' and 'metyrapone test' [12], one 'short synacthen test' [11], one 'modified low dose synacthen test' [6,13] and two used 'long-acting porcine sequence ACTH stimulation test' [9,14]. Details for cortisol stimulation technique used in each study are provided in Table 2 and short description of stimulation tests that were used in Table 3. Cortisol levels units were all converted to μg/dl for consistency purposes.<br><br>Prevalence of AI<br>As previously mentioned, the AI assessment techniques and the reference cortisol thresholds varied across the 11 studies included. Hence, the prevalence of AI couldn't be calculated cohesively, with some of the studies showing different results even between the two or three methods used in specific populations at the same time. Table 4 summarizes the AI prevalence reported in each study, considering the various assessment techniques. The highest AI prevalence (92.9%) was found by Hansen N et al., using 2h ACTH stimulation test [15], and the lowest (5.9%) by Moel DI et al., using 6h ACTH stimulation test [12], with both having examined patients with SSNS after the steroid treatment completion. In four studies, other steroid complications were recorded along with the AI quantification: obesity, hypertension, cushingoid appearance, hirsutism, myalgia - weakness and cataract. The number of children who developed signs of steroid toxicity (ST) compared to those diagnosed with AI (ST/AI) were 4/5 [19], 23 /28 [8], 23/AI 13 [6] and 28/36 [19] respectively.<br><br>AI variations among NS Subtypes and Associated Risk Factors	Page 5,6,7

PRISMA 2020 Checklist

Section and Topic	Item #	Checklist item	Location where item is reported
		The prevalence of AI among the different NS subtypes was assessed in six studies. Three research groups identified FRNS as the one most commonly associated with AI, with frequencies of 63.6% [7], 100% [12], and 77.8% [14] respectively. Moel DI et al. [12] and Mantan M et al. [8] showed that plasma cortisol was significantly lower in frequently relapsing individuals compared to the infrequently relapsers (p =<0.05). Jaiganesh M et al. reported IFRNS as the most frequent subtype (84.6%) [9], whereas Mantan M et al. found the highest AI prevalence in SRNS (57%) with FRNS to follow (28%) [8] and Abu Bakar K et al. in SDNS (76.9%) [6]. Three of the studies compared the relapses within the first year between the patients developed AI and those who did not, all showing a higher incidence of relapse in the AI group. Leisti S et al. noted that all the patients with AI belonged to the group whose first relapse occurred within a year (p<0.10) [7]. Similarly, Leisti S et al. [16] and Abeyagunawardena AS et al. [13] showed a significant difference in the prevalence of relapses during the first year between the AI and non-AI groups, with a 88% vs 79% (P < 0.05) and a 100% vs 58% (p=0.01) respectively.<br>In six of the eleven studies, risk factors associated with AI were recorded. Two studies found a significant correlation between AI and young age [6,7], while another with older age [9]. In two studies, higher/longer exposure to steroids was marked as a statistically significant risk factor for AI [8,14]. Table 5 provides a detailed summary of the risk factors, along with the most frequent NS subtypes associated with AI and the 12-month relapse incidence for both AI and non-AI patients across the reviewed studies.	
	20c	Present results of all investigations of possible causes of heterogeneity among study results.<br><br>Over the past 50 years, only a limited number of studies have focused on various aspects of the diagnosis and management of steroid-induced AI in children with NS. Despite five decades of sporadic investigation, most of the data have emerged only recently and remains inconclusive, underlying the need for standardized protocols and further research.	Page 7
	20d	Present results of all sensitivity analyses conducted to assess the robustness of the synthesized results.<br>Robustness not assessed due to missing outcomes/results	Page 4 - 7
Reporting biases	21	Present assessments of risk of bias due to missing results (arising from reporting biases) for each synthesis assessed.<br>Risk of bias not assessed due to missing outcomes/results	Page 4 - 7
Certainty of evidence	22	Present assessments of certainty (or confidence) in the body of evidence for each outcome assessed.<br>Certainty not assessed due to missing outcomes/results	Page 4 - 7
**DISCUSSION**			
Discussion	23a	Provide a general interpretation of the results in the context of other evidence.<br><br>The prevalence of AI in the population examined ranged widely from 5.9% to 92.9%, depending heavily on the diagnostic method and the timing of testing. Such disparity reflects a significant methodological heterogeneity among studies, with at least seven different diagnostic protocols used. These included the short synacthen test (SST), low-dose ACTH tests, and early morning cortisol measurements, each using different cut-off thresholds. For example, Hansen et al. reported the highest AI rate using a 2-hour ACTH stimulation test, while Moel et al. found the lowest prevalence using a 6-hour ACTH test. The choice of diagnostic test appears to substantially influence AI detection, a point emphasized by Jaiganesh et al., who noted that normal basal cortisol levels were not predictive of normal ACTH responses in nearly 77% of affected children.<br>This variability extends to the timing of testing, with studies assessing AI during ongoing steroid therapy, immediately after steroid discontinuation, or weeks thereafter. The influence of steroid pharmacokinetics and recovery dynamics of the hypothalamic-pituitary-adrenal (HPA) axis likely contributes to inconsistent detection rates. Furthermore, no universally accepted threshold for cortisol insufficiency was used across studies, complicating comparative analysis and pooled prevalence estimates.<br><br>The heterogeneity in AI prevalence is also mirrored in the differential risk across NS subtypes. While most studies focused exclusively on steroid-sensitive NS, some included children with steroid-resistant, frequently relapsing or steroid-dependent courses. Several studies identified FRNS as the subtype most associated with AI, with reported frequencies as high as 100% . Others found SDNS or SRNS to be the most affected, while IFRNS	Page 7-8

PRISMA

PRISMA 2020 Checklist

Section and Topic	Item #	Checklist item	Location where item is reported
		showed the lowest association. This inconsistency may be partially attributed to differences in patient selection, steroid dosing histories, and duration off therapy at the time of testing.	
	23b	Discuss any limitations of the evidence included in the review.<br><br>Despite growing interest in this topic, several key questions remain unanswered. First, there is no consensus on the optimal timing or preferred method for AI assessment in NS patients. Although morning cortisol has been proposed as the initial step for evaluating HPA axis integrity [3, 29], it appears to be inadequate as a standalone tool. Moreover, the ACTH stimulation tests used in clinical practice differ considerably in design, availability, and interpretive thresholds, and debate regarding which test most accurately detects AI is ongoing [3]. Second, the prevalence of AI across NS subtypes remains unclear due to inconsistent subgroup analysis and limited inclusion of SRNS, SDNS, and IFRNS patients. Third, the clinical significance of subclinical AI, particularly its impact on relapse risk and morbidity, needs to be better defined through prospective longitudinal studies.	Page 8-9
	23c	Discuss any limitations of the review processes used.<br>Quantitative Synthesis (Meta-analysis) not possible- studies not sufficiently similar in design, participants, interventions, and outcomes	Page 7-9
	23d	Discuss implications of the results for practice, policy, and future research.<br><br>Despite growing interest in this topic, several key questions remain unanswered. First, there is no consensus on the optimal timing or preferred method for AI assessment in NS patients. Although morning cortisol has been proposed as the initial step for evaluating HPA axis integrity, it appears to be inadequate as a standalone tool. Moreover, the ACTH stimulation tests used in clinical practice differ considerably in design, availability, and interpretive thresholds, and debate regarding which test most accurately detects AI is ongoing . Second, the prevalence of AI across NS subtypes remains unclear due to inconsistent subgroup analysis and limited inclusion of SRNS, SDNS, and IFRNS patients. Third, the clinical significance of subclinical AI, particularly its impact on relapse risk and morbidity, needs to be better defined through prospective longitudinal studies.<br>Also, strategies to minimize HPA axis suppression deserve further exploration. These include evaluating the safety and efficacy of alternate-day steroid regimens, early transition to steroid-sparing agents, and personalized tapering protocols. As emphasized in NICE guideline 2024, during the steroid tapering phase, when AI-related adverse events are most likely to occur, there is insufficient evidence on how best to taper in order to ensure timely recovery of adrenal function. Future studies should also examine the role of genetic and environmental factors in modulating steroid responsiveness and HPA recovery, as suggested by the differing outcomes of the PREDNOS 2 trial compared to South Asian studies.<br>Finally, joint Nephrology–Endocrinology guidelines are needed to provide standardized recommendations for AI evaluation and management in patients with NS. It is well known that during periods of adrenal insufficiency, patients are at increased risk of adrenal crisis, particularly during infections, major surgeries or other physiological stressors. The current treatment approach for any first episode of childhood NS includes at least 4 weeks of Prednisone / Prednisolone 60 mg/m^2/day, followed by quick tapering. This cumulative steroid exposure invariably meets the criteria for long-term glucocorticoid therapy (treatment duration >3–4 weeks with doses exceeding the physiologic cortisol secretion (hydrocortisone 10mg/m2/day or prednisolone 2.5mg/ m2/day). According to the recent joint clinical guideline from the European Society of Endocrinology and the Endocrine Society for glucocorticoid-induced adrenal insufficiency, all NS patients receiving treatment can be expected to have suppressed adrenal function. The guideline further recommends that all patients with current or recent glucocorticoid use who have not undergone biochemical testing to rule out glucocorticoid-induced AI should receive stress-dose coverage during periods of stress, along with appropriate patient education to raise awareness of when stress dosing is required. Further research is required to clarify whether cortisol replacement therapy should be initiated in confirmed AI, and to define the optimal dosing regimen and duration.<br><br>In light of the current evidence and the endocrine practice guidelines we propose a pragmatic approach (Table 6), that needs to be adjusted to the local protocols for AI management in children with NS.<br>Children with FRNS, SDNS, prolonged steroid exposure (>4 weeks), cumulative steroid doses exceeding the normal cortisol secretion (hydrocortisone 10mg/m2/day) or frequent relapses within the first year, are at high risk for AI and may undergo HPA axis evaluation. A morning serum cortisol level, measured between 8:00 and 9:00 AM, after holding glucocorticoid dose for at least 24 hours and only after reaching the range of a	Page 8-9

PRISMA 2020 Checklist

Section and Topic	Item #	Checklist item	Location where item is reported
		physiologic equivalent daily dose, seems to be a reasonable screening approach. An ACTH stimulation test may be further required when the cortisol level is low or indeterminate or in conditions that total serum cortisol is falsely low. Immediate initiation of stress-dose parenteral glucocorticoids and fluid resuscitation is recommended for children with confirmed AI or for those with current or recent glucocorticoid use who have not yet been tested for glucocorticoid-induced AI, during periods of acute illness, surgery, or trauma. Adrenal crisis might also occur in patients taking oral supraphysiologic doses of glucocorticoids, if drug availability suddenly decreases due to missed doses or gastroenteritis. Stress-dose steroids warrants consideration in patients presenting with hemodynamic instability, hypoglycemia or acute abdominal symptoms. It is advised that clinicians and families remain aware of the potential AI features and that rescue hydrocortisone for stress dosing (hydrocortisone 100mg/m2) be prescribed when appropriate. Physiologic daily dose replacement (hydrocortisone 8–10 mg/m^2/day in 2–3 divided doses) is advocated in all the patients with suspected AI until normal HPA function is demonstrated. In practice, this may require 3–6 months of follow-up, though individual recovery times vary. Reassessment could be scheduled every 1-3 months until recovery is documented. A final tapering phase with hydrocortisone which is preferred over prednisolone for its shorter half-life to facilitate faster recovery of adrenal function might be considered for frequent relapsers or steroid-dependent nephrotic patients. If symptoms of adrenal insufficiency occur, the glucocorticoid regimen could be reinstated and not discontinued until recovery of the HPA axis is documented. In these patients, early initiation of glucocorticoid-sparing agents (e.g., levamisole, ciclosporin) is encouraged to reduce cumulative steroid burden and mitigate AI risk.	
**OTHER INFORMATION**			
Registration and protocol	24a	Provide registration information for the review, including register name and registration number, or state that the review was not registered.<br><br>Kalliopi Vardaki, Maria Bitsori, Emmanouil Galanakis. Steroid Induced Adrenal Insufficiency in Children with Nephrotic Syndrome. PROSPERO 2025 CRD420251082353. Available from https://www.crd.york.ac.uk/PROSPERO/view/CRD420251082353.	NA
	24b	Indicate where the review protocol can be accessed, or state that a protocol was not prepared.<br><br>PROSPERO 2025 CRD420251082353. Available from https://www.crd.york.ac.uk/PROSPERO/view/CRD420251082353	NA
	24c	Describe and explain any amendments to information provided at registration or in the protocol.<br>Authors contributed to the systematic review - Ilianna Maniadaki is now added	NA
Support	25	Describe sources of financial or non-financial support for the review, and the role of the funders or sponsors in the review.<br>No financial support	NA
Competing interests	26	Declare any competing interests of review authors.<br>No competing interests	NA
Availability of data, code and other materials	27	Report which of the following are publicly available and where they can be found: template data collection forms; data extracted from included studies; data used for all analyses; analytic code; any other materials used in the review.<br><br>Data not publicly available – for any further information required, please contact the corresponding author (Maria Bitsori)	NA

From: Page MJ, McKenzie JE, Bossuyt PM, Boutron I, Hoffmann TC, Mulrow CD, et al. The PRISMA 2020 statement: an updated guideline for reporting systematic reviews. BMJ 2021;372:n71. doi: 10.1136/bmj.n71. This work is licensed under CC BY 4.0. To view a copy of this license, visit https://creativecommons.org/licenses/by/4.0/
